# The addition of CD38 monoclonal antibody to triplet regimens improves survival in newly diagnosed multiple myeloma with high-risk cytogenetics: a systematic review and meta-analysis of randomized controlled trials

**DOI:** 10.3389/fimmu.2025.1744165

**Published:** 2026-01-06

**Authors:** Bin Hu, Dan Fang, Ling Jiang, Tianqi Li, Kexia Chen, Jinxia Cao, Jun Wang

**Affiliations:** 1 Department of Hematology, Changde Hospital, Xiangya School of Medicine, Central South University (First People’s Hospital of Changde City), Changde, China; 2 Department of Oncology, Changde Hospital, Xiangya School of Medicine, Central South University (First People’s Hospital of Changde City), Changde, China

**Keywords:** CD38 monoclonal antibodies, high-risk cytogenetics, meta-analysis, multiple myeloma, quadruplet regimens

## Abstract

**Background:**

The efficacy of CD38 monoclonal antibody (mAb)-based quadruplet regimens versus triplet regimens in newly diagnosed multiple myeloma (NDMM) patients with high-risk cytogenetics remains controversial. This meta-analysis aims to consolidate evidence from randomized controlled trials (RCTs) to resolve this clinical uncertainty.

**Methods:**

We systematically searched PubMed, EMBASE, and the Cochrane Library for RCTs comparing CD38 mAb-based quadruplet regimens with triplet regimens in NDMM patients with high-risk cytogenetics. The primary outcomes were the rate of minimal residual disease (MRD) negativity at a sensitivity of 10^-5^ and progression-free survival (PFS).

**Results:**

Nine RCTs comprising 4557 patients were included. Compared to triplet regimens, CD38 mAb-based quadruplet regimens were associated with a significantly higher rate of MRD negativity (pooled OR = 2.02, 95% CI: 1.41-2.88, P = 0.0001) and a significantly improved PFS (pooled HR = 0.74, 95% CI: 0.59-0.94, P = 0.01). However, subgroup analyses revealed that the PFS benefit was not significant for isatuximab-based quadruplet regimens (pooled HR = 1.04, 95% CI: 0.67-1.62, P = 0.84) or in transplant-ineligible patients (pooled HR = 0.79, 95% CI: 0.56-1.13, P = 0.19).

**Conclusion:**

The incorporation of CD38 mAbs, particularly daratumumab, into triplet regimens improves depth of response and PFS in NDMM patients with high-risk cytogenetics.

**Systematic review registration:**

https://inplasy.com/inplasy-2025-10-0103/

, identifier INPLASY2025100103.

## Introduction

For patients with newly diagnosed multiple myeloma (NDMM), a triplet regimen consisting of a proteasome inhibitor, an immunomodulatory agent, and dexamethasone was the standard induction therapy (1). CD38 monoclonal antibodies (mAbs), such as daratumumab and isatuximab, exert their effects through multiple mechanisms, including Fc-dependent immune effector activity, direct pro-apoptotic effects, and immunomodulation via the elimination of CD38-expressing immunosuppressive cells (2–4). These multifaceted actions contribute to their significant cytotoxicity against multiple myeloma (MM) cells (5). The addition of a CD38 mAb to a backbone triplet regimen has led to the establishment of quadruplet therapies, which have now become widely adopted as induction therapy for NDMM (6). The phase 3 PERSEUS study demonstrated that adding daratumumab to bortezomib-lenalidomide-dexamethasone (VRd) induction significantly improved progression-free survival (PFS) in patients with NDMM (hazard ratio [HR] for disease progression or death = 0.42; 95% confidence interval [CI]: 0.30-0.59; P<0.001) (7). Similarly, the phase 3 IMROZ trial showed that first-line isatuximab in combination with VRd significantly improved PFS (HR = 0.6, 98.5% CI: 0.41-0.88; p<0.001) and induced deeper responses compared to VRd alone (8, 9). Collectively, this clinical evidence has established CD38 mAb-based quadruplet regimens as the new standard induction therapy, superseding conventional triplet regimens (10).

Although the addition of CD38 mAbs to triplet regimens is associated with longer survival, this benefit is not consistent across all subgroups, particularly in NDMM patients with high-risk cytogenetics, a category which the 2016 International Myeloma Working Group (IMWG) consensus defined by identifying del(17p), t(4,14), and t(14,16) as primary lesions while also recognizing gain(1q) associated with del(1p) carrying poor risk (11). The 2025 IMWG consensus introduces a more sophisticated combinatorial definition of high-risk disease. Key changes include a stricter cutoff for del(17p) and, most notably, the reclassification of IgH translocations [t(4,14), t(14,16), or t(14,20)] to the high risk tier only when they co-occur with gain(1q) and/or del(1p32). The presence of monoallelic del(1p32) along with gain(1q) or biallelic del(1p32) is directly designated as high-risk cytogenetics (12). Among the currently reported clinical studies in NDMM patients with high-risk cytogenetics, the phase 3 PERSEUS trial (7) stands out as the only one to demonstrate that the addition of daratumumab significantly improved PFS. In contrast, other trials, including ALCYONE (13–15), AMaRC 03-16 (16), CASSIOPEIA (17–19), CEPHEUS (20), GRIFFIN (21–23), and OCTANS (24, 25), found that the addition of daratumumab did not confer a significant PFS benefit in these patients. Moreover, in both the GMMG-HD7 (26, 27) and IMROZ (8, 9) trials, the isatuximab-based quadruplet regimen did not demonstrate superior PFS compared to triplet therapy in the high-risk cytogenetic subgroup.

The optimal induction therapy for NDMM patients with high-risk cytogenetics remains controversial, specifically whether a CD38 mAb-based quadruplet regimen or a conventional triplet regimen is preferable. Aggregating data from multiple randomized controlled trials (RCTs) through a meta-analysis represents a robust approach to resolve this ongoing debate. Therefore, we conducted this meta-analysis to systematically compare the efficacy of CD38 mAb-based quadruplet regimens versus triplet therapies in this patient population.

## Materials and methods

### Search strategy

Two independent investigators systematically performed literature searches across multiple electronic databases, including PubMed, EMBASE, and the Cochrane Library. The inclusion criteria were restricted to published RCTs with accessible full-text articles. Non-English studies, unpublished or ongoing trials were excluded in our study. To ensure comprehensive coverage, the reference lists of all eligible publications were manually examined to identify other potentially relevant studies. The search encompassed all available literature published through October 2025. The complete search methodology is detailed in the Supplementary information.

### Selection criteria

The identified studies underwent independent assessment by two reviewers. Studies were included if they met the following criteria:

- research design: RCTs;
- participants: NDMM patients with high-risk cytogenetics;
- intervention: CD38 mAb-based quadruplet regimens versus triplet regimens;
- outcomes: the rate of negative status for MRD (10–^5^ threshold) and PFS.

### Data extraction

Two reviewers independently executed data extraction for the included RCTs, covering detailed medication schedules, drug dosages, cytogenetic risks related data (criteria for high-risk cytogenetics, cutoffs, and methods), MRD related data (time point, sample source, methods, and sensitivity), and survival outcomes.

### Methodological quality appraisal

Two independent reviewers evaluated the methodological quality of all included studies. The Cochrane Collaboration’s Risk of Bias tool (28) was utilized to assess the quality of the randomized controlled trials.

### Outcomes assessments

The objective was to compare the negative MRD status rate and PFS between the two arms.

### Statistical analysis

We utilized RevMan 5.4 for analyses. Study heterogeneity was examined with the I² statistic, which interpreted values of 25%-50% as low, 50%-75% as moderate, and >75% as high. Given the anticipated clinical and methodological diversity across the included trials, the random-effects model was pre-specified as our primary analytical framework. This model provides a more conservative and generalizable estimate by accounting for both within-study and between-study variability. A fixed-effect model was only considered appropriate in scenarios where both the I² statistic indicated negligible heterogeneity (I² ≤ 25%) and the studies were qualitatively judged to be highly homogeneous in their design and patient cohorts.

## Results

### Selection of the trials

**Figure 1:**
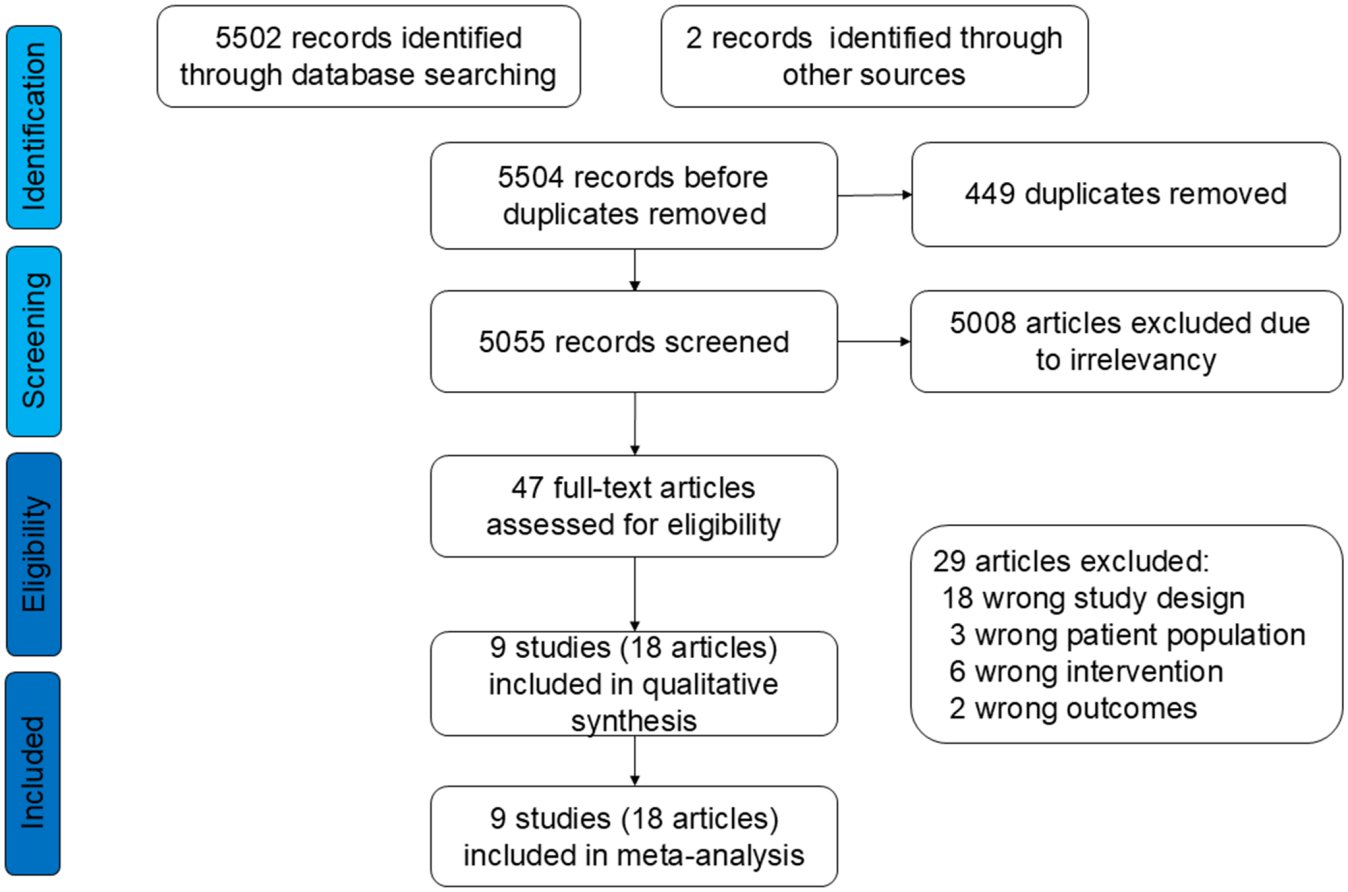
Flowchart of literature search and study selection.

The protocol for identifying, screening, and selecting relevant literature is illustrated in **Figure 1**. The systematic search initially identified 5504 records. Through the application of inclusion criteria, 18 publications from 9 randomized controlled trials (RCTs) were selected. This resulted in a meta-analysis cohort comprising 4,557 patients.

### Characteristics of the trials

**Figure 2:**
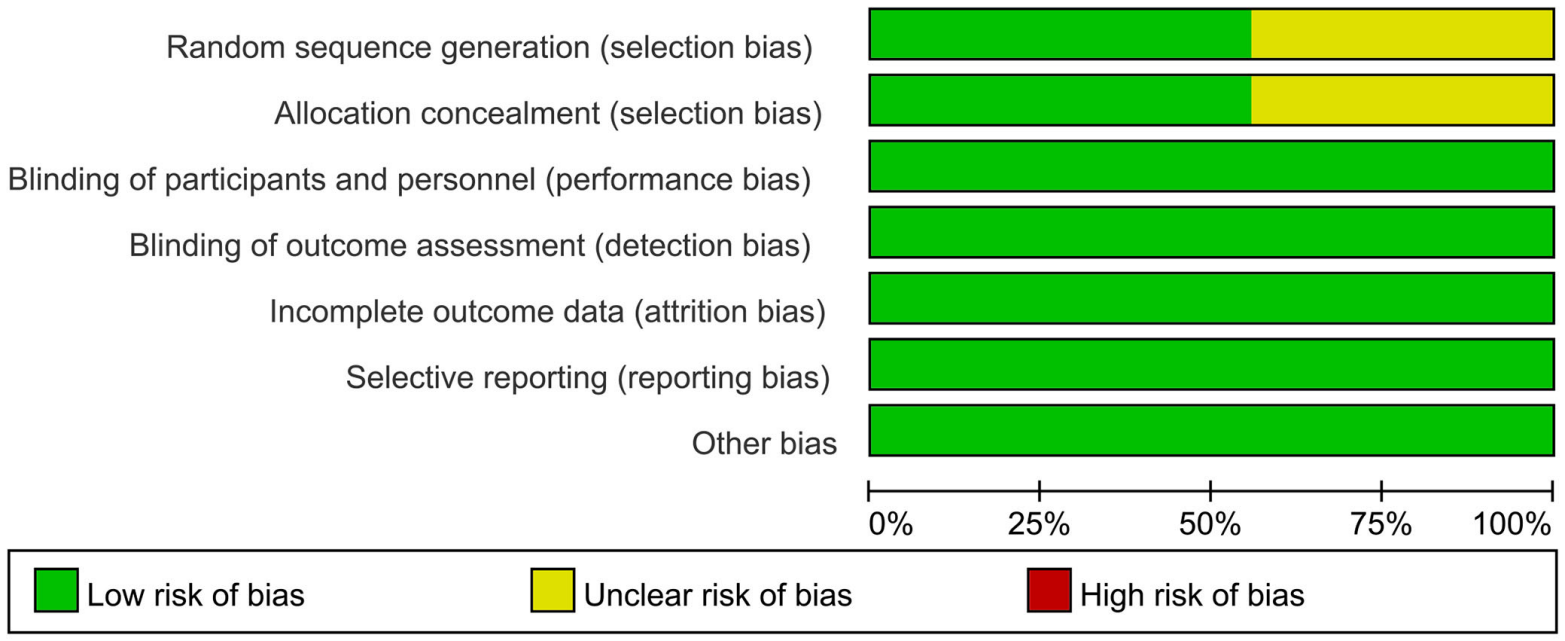
Risk of bias summary for RCTs.

**Figure 3:**
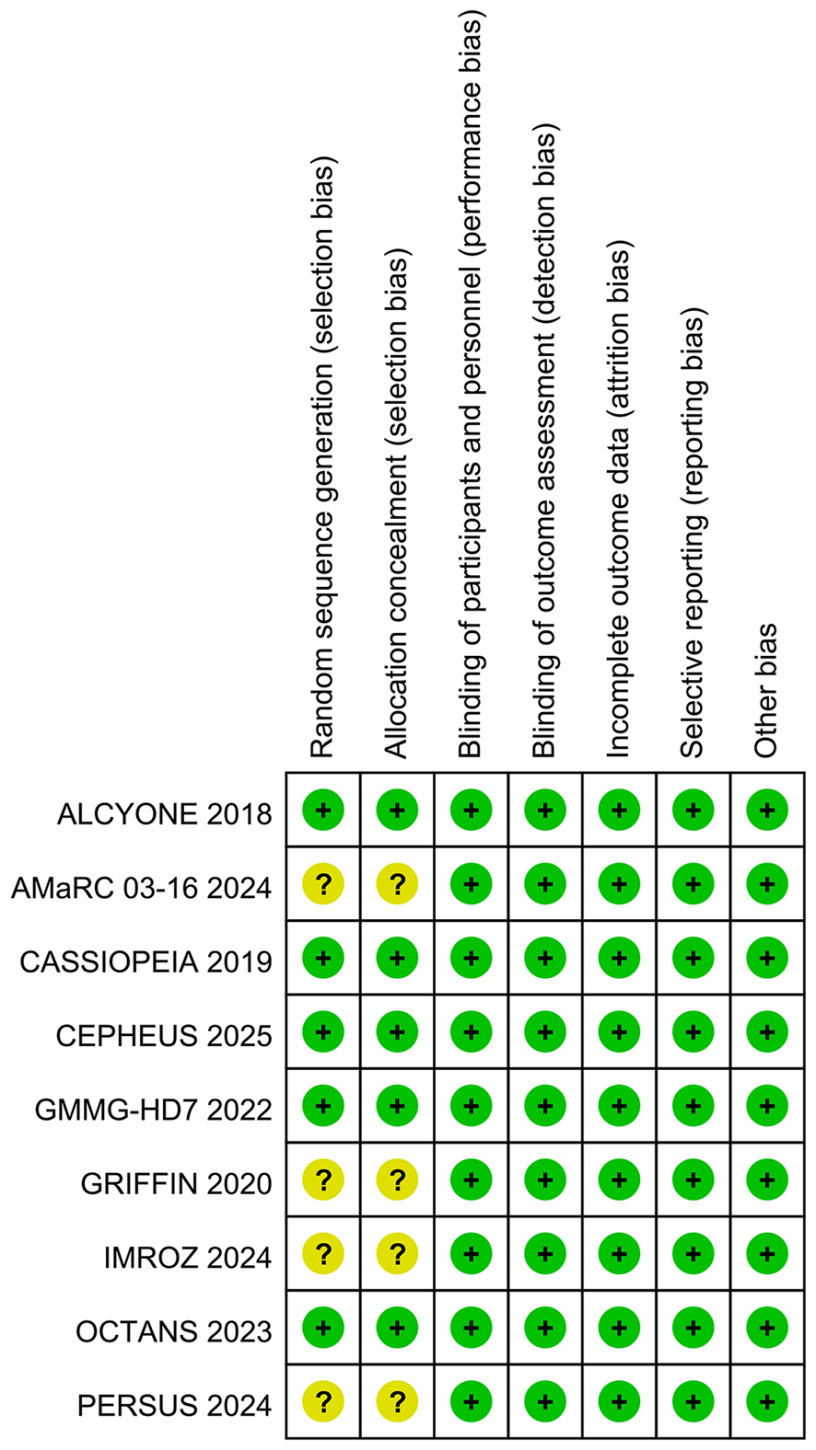
Risk of bias graph for RCTs.

**Table 1** presents the primary characteristics of the 9 RCTs. **Table 2** displays the criteria for high-risk cytogenetics in each RCT. Each study had a full-text article available, and all RCTs included in the analysis were assessed as high quality. The quality appraisal of the 9 RCTs is shown in **Figures 2** and **3**. The basis for the risk of bias assessment for each RCT is provided in **Supplementary Table 1**.

**Table 1:** Attributes of studies meeting the inclusion criteria for the meta-analysis.

Clinical trials	Year	Number of patients (all/high-risk)	Median age (y)	Aera	Patients	Study arms		Study design
						Quadruplet regimens	Triplet regimens	
ALCYONE	2018	706/98	71	Global	transplant-ineligible NDMM	D-VMP	VMP	phase 3 RCT
AMaRC 03-16	2024	129/19	75	Australia	transplant-ineligible NDMM	D-VCD	VCD	phase 2 RCT
CASSIOPEIA	2019	1085/168	59	European	transplant-eligible NDMM	D-VTd	VTd	phase 3 RCT
CEPHEUS	2025	395/52	70	Global	transplant-ineligible or transplant-deferred NDMM	D-VRd	VRd	phase 3 RCT
GMMG-HD7	2022	660/124	59.5	Germany	transplant-eligible NDMM	Isa-VRd	VRd	phase 3 RCT
GRIFFIN	2020	207/30	60	United States	transplant-eligible NDMM	D-VRd	VRd	phase 2 RCT
IMROZ	2024	446/74	72	Global	transplant-ineligible NDMM	Isa-VRd	VRd	phase 3 RCT
OCTANS	2023	220/48	69	China	transplant-ineligible NDMM	D-VMP	VMP	phase 3 RCT
PERSEUS	2024	709/154	60	Europe and Australia	transplant-eligible NDMM	D-VRd	VRd	phase 3 RCT

NDMM, newly diagnosed multiple myeloma; D, daratumumab; VMP, bortezomib, melphalan and prednisone; RCT, randomized controlled trial; VCD, bortezomib, cyclophosphamide and dexamethasone; VTd, bortezomib, thalidomide and dexamethasone; VRd, bortezomib, lenalidomide, and dexamethasone; Isa, isatuximab.

**Table 2:** Criteria for high-risk cytogenetics in each RCT.

Clinical trials/year	Criteria for high-risk cytoge- netics	Cutoffs	Methods
ALCYONE 2018	del(17p) and/or t(4,14) and/or t(14,16)	–	FISH or karyotype testing
AMaRC 03-16 2024	del(17p) and/or t(4,14) and/or t(14,16)	–	FISH
CASSIOPEIA 2019	del(17p) and/or t(4,14)	del17p (≥50% abnormal cells) and t(4,14) (≥30% abnormal cells)	FISH
CEPHEUS 2025	del(17p) and/or t(4,14) and/or t(14,16)	–	FISH
GMMG-HD7 2022	del(17p) and/or t(4,14) and/or t(14,16)	≥10% abnormal cells	–
GRIFFIN 2020	del(17p) and/or t(4,14) and/or t(14,16)	–	FISH
IMROZ 2024	del(17p) and/or t(4,14) and/or t(14,16)	del17p (≥50% abnormal cells), t(4,14) and t(14,16) (≥30% abnormal cells)	FISH
OCTANS 2023	del(17p) and/or t(4,14) and/or t(14,16)	–	FISH or karyotype analysis
PERSEUS 2024	del(17p) and/or t(4,14) and/or t(14,16)	–	FISH

FISH, fluorescence *in situ* hybridization.

### MRD-negative rate

**Figure 4:**
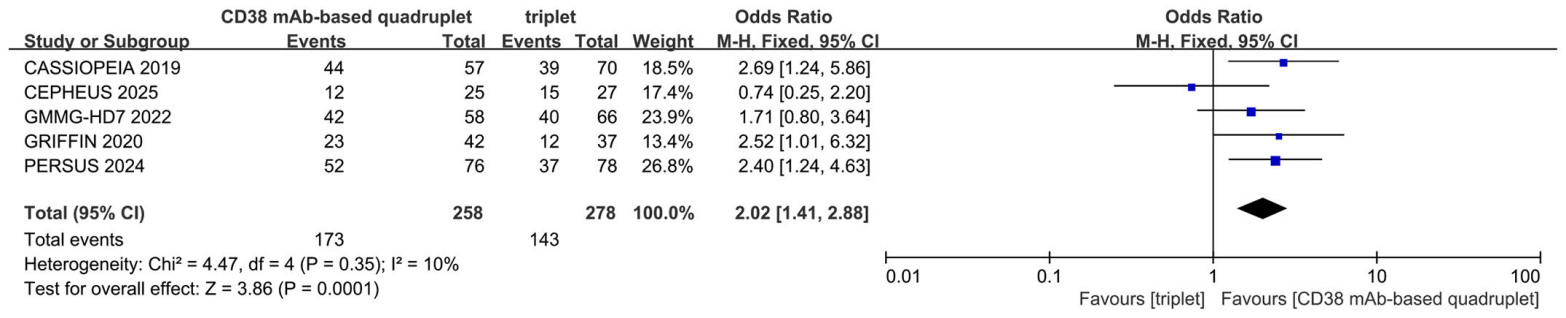
Forest plot of MRD negativity in CD38 mAb-based quadruplet regimens versus triplet regimens.

Five studies were included in the analysis. The MRD assessment in the 5 RCTs is displayed in **Supplementary Table 2**. Compared with triplet regimens, CD38 mAb-based quadruplet regimens achieved a significantly higher MRD-negative rate (pooled OR = 2.02, 95% CI: 1.41-2.88, P = 0.0001; low heterogeneity, P = 0.35, I² = 10%; **Figure 4**).

### PFS

**Figure 5:**
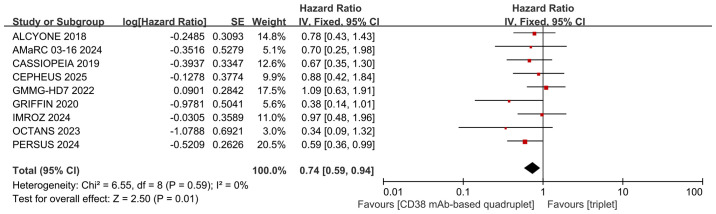
Forest plot of PFS in CD38 mAb-based quadruplet regimens versus triplet regimens.

Nine studies were included in the analysis. Compared with triplet regimens, CD38 mAb-based quadruplet regimens achieved significantly improved PFS (pooled HR = 0.74, 95% CI: 0.59-0.94, P = 0.01), with no heterogeneity (P = 0.59, I² = 0%; **Figure 5**).

### Subgroup analysis

**Figure 6:**
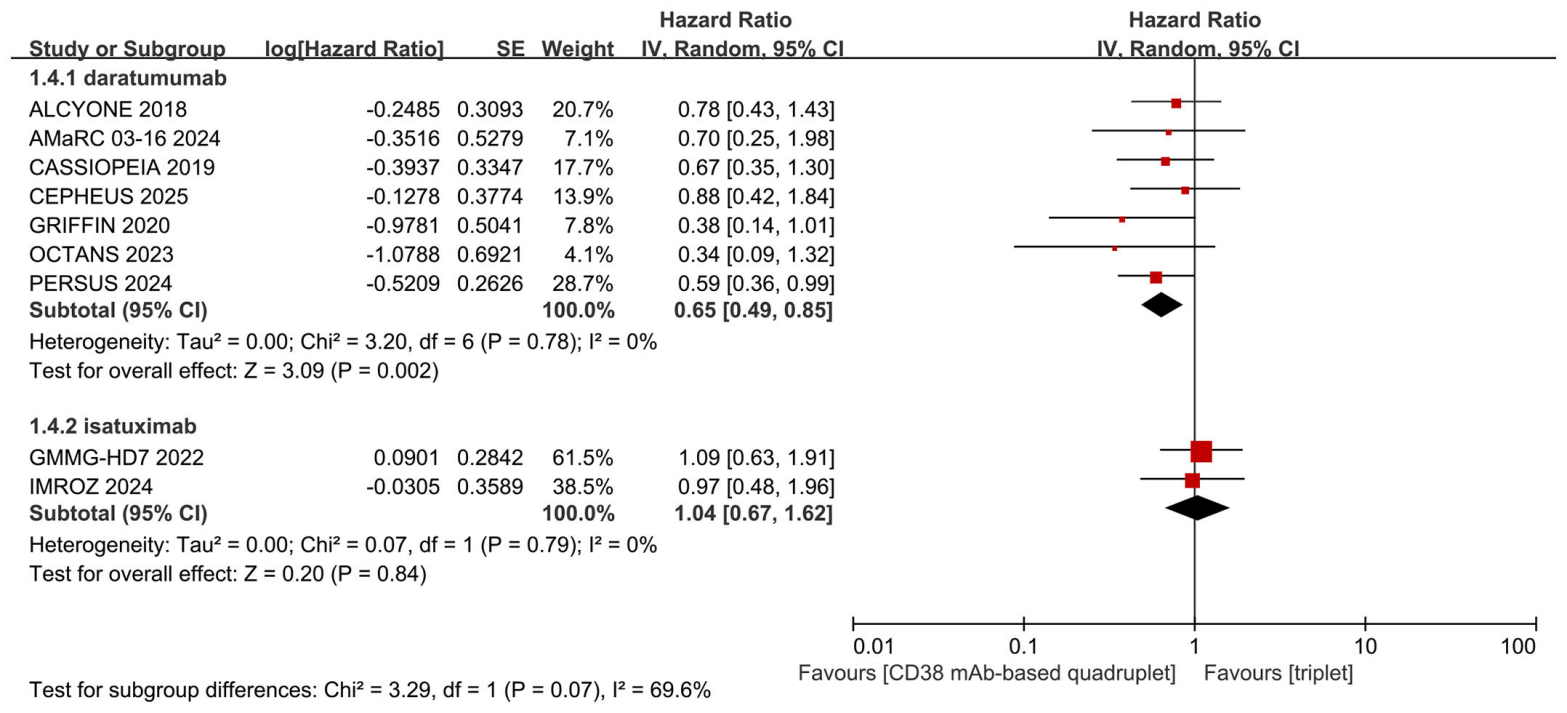
Subgroup analyses for PFS regarding type of CD38 mAbs (daratumumab or isatuximab).

**Figure 7:**
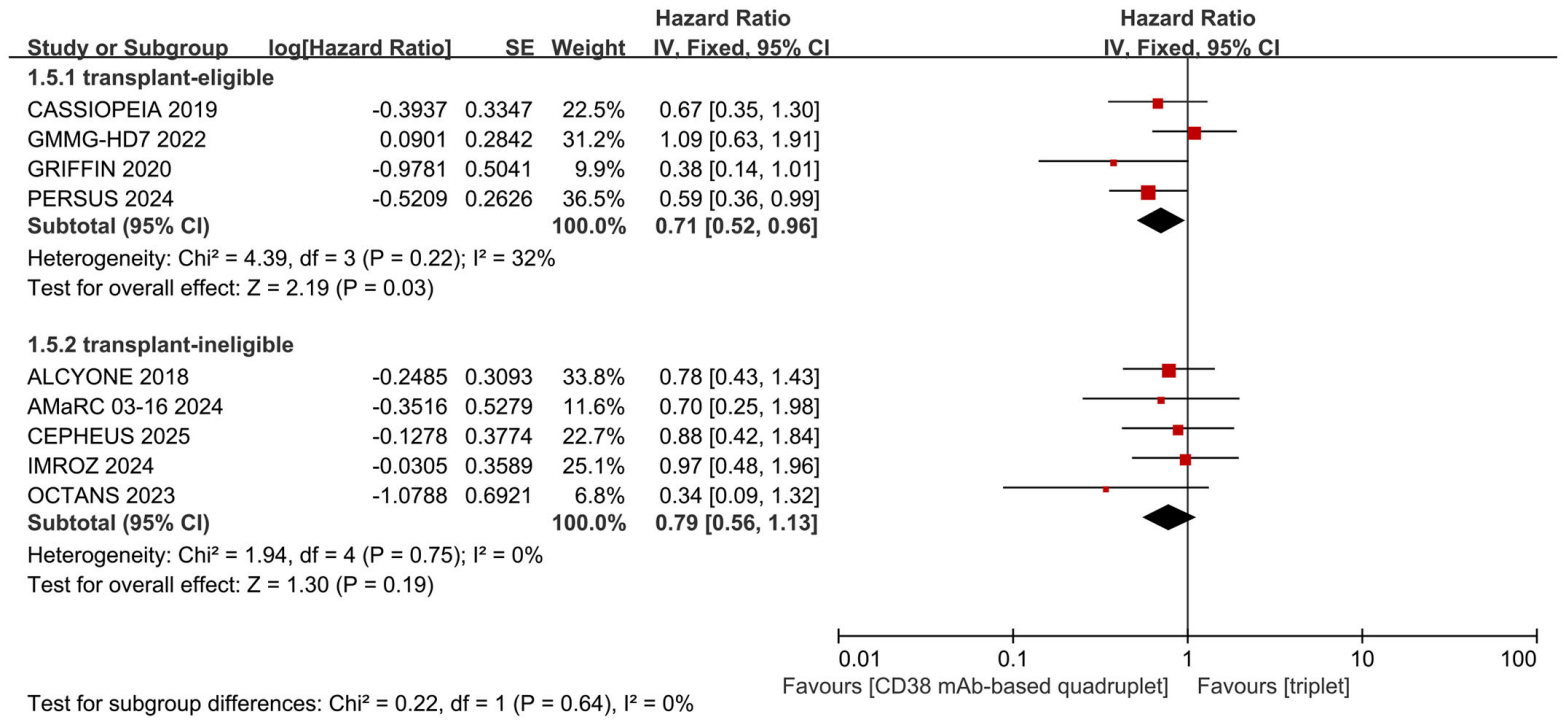
Subgroup analyses for PFS regarding transplant eligibility (transplant-eligible or ineligible).

We performed subgroup analyses of PFS based on the type of CD38 mAbs (daratumumab or isatuximab) and transplant eligibility (transplant-eligible or ineligible). The detailed data are shown in **Figures 6** and **7**. Differing results were observed in the isatuximab subgroup and transplant-ineligible subgroup. The isatuximab-incorporated quadruplet regimens did not achieve a better PFS (pooled HR = 1.04, 95% CI: 0.67-1.62, P = 0.84; no heterogeneity, P = 0.79, I² = 0%) than triplet regimens in NDMM with high-risk cytogenetic factors. For transplant-ineligible NDMM patients with high-risk cytogenetics, the incorporation of a CD38 monoclonal antibody into a triplet regimen did not confer a superior PFS advantage over triplet regimens alone (pooled HR = 0.79, 95% CI: 0.56-1.13, P = 0.19; no heterogeneity, P = 0.75, I² = 0%).

### Heterogeneity analysis

Heterogeneity analysis was performed for the two primary outcomes. No statistically significant heterogeneity was detected. The results are shown in **Figures 4** and **5**.

### Sensitivity analysis

We performed sensitivity analyses to assess the impact of excluding individual studies or specific groups of studies (such as studies not exclusively using NGS for MRD detection, phase 2 RCTs, or studies with non-immunomodulatory drugs backbones) on the pooled outcomes. The pooled estimate for the MRD−negative rate remained robust and was not substantially altered by the exclusion of any single study or the specified study groups. However, when the PERSEUS trial was excluded, the difference in PFS between the treatment arms was no longer statistically significant (pooled HR = 0.79, 95% CI: 0.61-1.02, P = 0.07). The detailed results are provided in **Supplementary Tables 3**-**6**.

## Discussion

The quadruplet regimen, which combines a CD38 mAb with a traditional triplet therapy, such as daratumumab-VRd(D-VRd), daratumumab-bortezomib-melphalan-prednisone(D-VMP), daratumumab-bortezomib-thalidomide-dexamethasone(D-VTd), daratumumab-bortezomib-cyclophosphamide-dexamethasone(D-VCD), and isatuximab-VRd(Isa-VRd), has become the standard of care for NDMM (10). These CD38 mAb-based quadruplet regimens have demonstrated deeper responses and longer survival compared to triplet regimens alone, with an adverse events profile considered acceptable (29, 30). However, whether the addition of a CD38 mAb benefits all NDMM patient subgroups, such as those with high-risk cytogenetics, remains controversial. Only the PERSEUS study (7) demonstrated a better PFS (HR 0.59, 95% CI 0.36-0.99) in the D-VRd group compared to VRd in NDMM with high-risk cytogenetics. Other studies have not shown a significant PFS benefit for CD38 mAb-based quadruplet regimens over triplets. This is evidenced by the following trials: ALCYONE (D-VMP vs. VMP; HR 0.78, 95% CI 0.43-1.43) (13–15), AMaRC 03-16 (D-VCD vs. VCD; HR 0.70, 95% CI 0.25-1.98) (16), CASSIOPEIA (D-VTd vs. VTd; HR 0.67, 95% CI 0.35-1.30) (17–19), CEPHEUS (D-VRd vs. VRd; HR 0.88, 95% CI 0.42-1.84) (20), GRIFFIN (D-VRd vs. VRd; HR 0.38, 95% CI 0.14-1.01) (21–23), OCTANS (D-VMP vs. VMP; HR 0.34, 95% CI 0.09-1.32) (24, 25), GMMG-HD7 (Isa-VRd vs. VRd; HR 1.09, 95% CI 0.63-1.91) (26, 27), IMROZ (Isa-VRd vs. VRd; HR 0.97, 95% CI 0.48-1.96) (8, 9). To address this controversial issue, we performed a meta-analysis of the aforementioned clinical trials. The pooled results confirmed that, compared to triplet regimens, CD38 mAb-based quadruplet regimens achieved significantly higher rates of MRD negativity and PFS in NDMM with high-risk cytogenetics. Our findings provide evidence to support the selection of CD38 mAb-based quadruplet regimens as first-line induction therapy for NDMM with high-risk cytogenetics.

The mechanisms of action of CD38 mAbs are multifactorial (31, 32). In 2008, daratumumab, a fully human immunoglobulin G-κ (IgG-κ) antibody, became the first CD38-targeting agent to be administered to patients with MM. In addition to its distinctive capacity to induce complement-dependent cytotoxicity (CDC), daratumumab eliminates MM cells through antibody-dependent cellular cytotoxicity (ADCC) and phagocytosis (ADCP) (3, 33). Similarly, isatuximab, a chimeric IgG-κ CD38 antibody, has multiple mechanisms of action including CDC, ADCC, and ADCP (34). Isatuximab exhibits weaker CDC and ADCP than daratumumab, while it possesses strong pro-apoptotic activity independent of cross-linking and inhibits CD38 ectoenzyme function (34). The bone marrow microenvironment (BMME) is crucial for MM pathogenesis and treatment (35, 36). A common feature of all immunosuppressive cells, including regulatory B cells (Bregs) and tumor-associated macrophages (TAMs), is the expression of high levels of CD38, which can be targeted by CD38 mAbs (35, 36). Treatment with daratumumab or isatuximab can rapidly deplete CD38+ regulatory T cells (Tregs), myeloid-derived suppressor cells (MDSCs), and Bregs and is associated with clonal expansion of CD4+ and CD8+ T cells in myeloma patients. Hence, CD38 mAbs therapy, besides targeting CD38-positive myeloma cells, can also restore an immunologically functional BMME exerting appropriate anti-MM T-cell responses (35, 36). Our study revealed that among NDMM with high-risk cytogenetics, daratumumab-based quadruplet regimens achieved deeper response and improved PFS compared with triplet regimens. In contrast, isatuximab-based quadruplets did not demonstrate a similar advantage, which may be attributed to their distinct mechanisms of action or the limited number of studies (only 2 RCTs) included in the isatuximab subgroup. Moreover, the lack of benefit observed in the isatuximab subgroup may also be related to specific design features of the included clinical trials. In the GMMG-HD7 trial (26, 27), the absence of consolidation therapy following the 18-week induction and autologous stem cell transplantation may have compromised treatment efficacy in high-risk cytogenetic MM patients. In the IMROZ study (8, 9), the interpretation of outcomes may potentially have been influenced by the protocol-permitted crossover of some control patients from Rd to isatuximab-Rd maintenance during the continuous treatment phase.

The administration of quadruplet regimens may raise toxicity concerns in specific patient populations, including those aged ≥65 years, individuals under 65 with significant comorbidities, and patients classified as frail according to the simplified IMWG (sIMWG) frailty score (37)—collectively representing the transplant-ineligible population. Older and frail patients are characterized by distinct tumor biology, altered pharmacokinetics, and diminished organ function; they experience more significant treatment-related toxicity (37). Consequently, a careful balance between efficacy and safety is crucial in managing transplant-ineligible individuals. Subgroup analysis in this study revealed that the addition of CD38 mAbs to triplet regimens did not improve PFS (pooled HR = 0.79, 95% CI: 0.56-1.13, P = 0.19) in transplant-ineligible patients. These results suggest that for transplant-ineligible NDMM patients with high-risk cytogenetics, the PFS benefit of CD38 mAb-based quadruplet regimens over triplet therapy remains uncertain. We attempted to compare the incidence of adverse events between the two groups in the transplant-ineligible subgroup to determine whether the lack of PFS benefit was due to diminished efficacy or treatment-related complications leading to dose modifications or early discontinuation. However, the absence of source data precluded this comparison, which constitutes a significant limitation of our study.

When interpreting the MRD results, it should be noted that there is heterogeneity in the assessment methodologies among the five included studies. As shown in **Supplementary Table 2**, although MRD assessments in all studies were conducted after consolidation therapy and during the maintenance phase, the specific timing of evaluation still varied across studies. In addition, the detection methods differed, employing either next-generation sequencing or flow cytometry. While these differences may introduce heterogeneity, our sensitivity analysis confirmed that the pooled MRD negativity rate remained robust and was not significantly influenced by any single study or by the different MRD detection techniques used, thereby strengthening the conclusion that quadruplet regimens significantly deepen treatment response.

An important consideration in interpreting our results is the heterogeneity in the definition of high-risk cytogenetics across the included trials, as summarized in **Table 2**. While most trials defined high-risk by the presence of del(17p), t(4,14), and/or t(14,16), the specific cutoffs for the percentage of abnormal plasma cells varied (e.g., ≥50% for del(17p) in CASSIOPEIA and IMROZ versus ≥10% in GMMG-HD7). Furthermore, the CASSIOPEIA trial notably excluded patients with t(14,16). A more rigorous approach would apply a uniform high-risk definition to reclassify patients or perform separate analyses for distinct cytogenetic subgroups. However, this was unfortunately not feasible for the present study as it requires access to individual patient data, which were not available to us. Despite these methodological differences, our sensitivity analysis confirmed that the exclusion of any single trial, including CASSIOPEIA, did not substantially alter the pooled PFS result. This consistency reinforces the robustness of our primary finding that CD38 mAb-based quadruplet regimens confer a PFS benefit in this clinically challenging population. Another limitation is that the included RCTs did not analyze patients with combinations like gain(1q) plus other cytogenetic abnormalities [such as t(4,14), t(14,16), t(14,20), or del(1p32)] within their high-risk cohorts, despite the 2025 IMWG consensus (12) categorizing such profiles as high-risk. Consequently, the direct relevance of our findings for guiding therapy in patients classified as high-risk under the latest criteria, especially those with gain(1q), remains uncertain.

It is noteworthy that the reported PFS benefit was heavily influenced by the PERSEUS trial, a finding corroborated by the sensitivity analysis, in which the exclusion of this single dataset led to a loss of statistical significance. The pronounced benefit observed in PERSEUS may be attributed to specific regimen (subcutaneous daratumumab-VRd), patient population (exclusively transplant-eligible with relatively lower median age) and its large sample size (which included 154 high-risk NDMM patients). The fact that the statistical significance of the pooled PFS benefit was lost upon the exclusion of the PERSEUS trial underscores that our global conclusion is fragile and may not reflect a consistent treatment effect across all studies. While the direction of the effect across trials is generally favorable, the current evidence does not permit a definitive conclusion of universal superiority for CD38 mAb-based quadruplet regimens in all high-risk NDMM settings. The observed benefit appears to be most robust and pronounced in the context of the D-VRd regimen for transplant-eligible patients, as exemplified by the PERSEUS trial.

The absence of a comparative analysis for overall survival (OS) constitutes a limitation of our study. Although we sought to compare OS between the two groups, only the ALCYONE trial among the nine included studies provided OS data specifically for the high-risk cytogenetic subpopulation, which precluded a formal meta-analysis. While the observed improvement in PFS is a validated and important intermediate endpoint, it does not invariably translate into an OS benefit. This is particularly relevant in the contemporary treatment landscape, where the availability of numerous effective salvage therapies, including bispecific antibodies and chimeric antigen receptor T-cell therapies, can substantially prolong survival after initial disease progression (38). Therefore, our findings of improved PFS with CD38 mAb-based quadruplet regimens, although encouraging, should be interpreted with caution regarding their ultimate impact on long-term survival.

Notably, this study relies heavily on hazard ratios and odds ratios reported in the original trials; however, in several instances, data for high-risk subgroups were derived from *post-hoc* or exploratory analyses. These estimates are inherently less reliable than pre-specified endpoints, which may diminish the robustness of our conclusions.

The results of this meta-analysis hold direct implications for clinical practice, yet their implementation must be carefully considered within real-world contexts. The significant improvements in PFS and MRD negativity support the consideration of CD38 mAb-based quadruplet regimens, particularly those incorporating daratumumab, as a preferred first-line strategy for NDMM patients with high-risk cytogenetics, which is consistent with current guideline recommendations. However, the absence of a demonstrated OS benefit in the available data, coupled with the substantial economic burden and variable accessibility of CD38 monoclonal antibodies across healthcare systems, presents a challenge to their universal adoption. In resource-limited settings, the decision to implement quadruplet therapy must involve a critical appraisal of the incremental efficacy—currently measured by intermediate endpoints—against the considerable financial cost. Therefore, while this evidence strengthens the efficacy profile of these regimens, it underscores the need for individualized treatment decisions that integrate patient-specific factors, local economic realities, and a cautious interpretation of the survival outcomes.

## Conclusion

The incorporation of CD38 mAbs, particularly daratumumab, into triplet regimens improves depth of response and PFS in NDMM patients with high-risk cytogenetics.

## Data Availability

The raw data supporting the conclusions of this article will be made available by the authors, without undue reservation.
