# Supplementary material for: The addition of CD38 monoclonal antibody to triplet regimens improves survival in newly diagnosed multiple myeloma with high-risk cytogenetics: a systematic review and meta-analysis of randomized controlled trials
Source: Front Immunol. 2026 Jan 6;16:1744165. doi: 10.3389/fimmu.2025.1744165 (PMC12816236; doi:10.3389/fimmu.2025.1744165)
Supplement: Supplementary file 1 [file Supplementaryfile1.docx]

Supplementary Table 1. The basis for the risk of bias assessment for each RCT.

| Studys | Random sequence generation | Allocation concealment | Blinding of participants and personnel | Blinding of outcome assessment | Incomplete outcome data | Selective reporting | Other bias |
| --- | --- | --- | --- | --- | --- | --- | --- |
| ALCYONE  2018 | Patients were randomly assigned by means of an interactive Web-response system. | Patients were randomly assigned by means of an interactive Web-response system. | Open-label | Open-label | The study protocol is available, with all prespecified outcomes reported. | No unexpected missing data for the outcomes | No other significant biases |
| AMaRC 03-16  2024 | No sufficient information to assess. | No sufficient information to assess. | Open-label | Open-label | The study protocol is available, with all prespecified outcomes reported. | No unexpected missing data for the outcomes | No other significant biases |
| CASSIOPEIA  2019 | Patients were randomly assigned by use of  an interactive web-based system. | Patients were randomly assigned by use of  an interactive web-based system. | Open-label | Open-label | The study protocol is available, with all prespecified outcomes reported. | No unexpected missing data for the outcomes | No other significant biases |
| CEPHEUS  2025 | Patients were randomly assigned by randomly permuted blocks using an interactive web-response system. | Patients were randomly assigned by randomly permuted blocks using an interactive web-response system. | Open-label | Open-label | The study protocol is available, with all prespecified outcomes reported. | No unexpected missing data for the outcomes | No other significant biases |
| GMMG-HD7  2022 | Randomisation was performed with a web-based system and balanced by using permuted blocks (block size of six) | Randomisation was performed with a web-based system and balanced by using permuted blocks (block size of six) | Open-label | Open-label | The study protocol is available, with all prespecified outcomes reported. | No unexpected missing data for the outcomes | No other significant biases |
| GRIFFIN  2020 | No sufficient information to assess | No sufficient information to assess | Open-label | Open-label | The study protocol is available, with all prespecified outcomes reported. | No unexpected missing data for the outcomes | No other significant biases |
| IMROZ  2024 | The specific randomization methodology and tools utilized were not delineated. | The specific randomization methodology and tools utilized were not delineated. | Open-label | Open-label | The study protocol is available, with all prespecified outcomes reported. | No unexpected missing data for the outcomes | No other significant biases |
| OCTANS  2023 | Patients were randomized by means of a computer-generated randomization schedule. | Patients were randomized by means of a computer-generated randomization schedule. | Open-label | Open-label | The study protocol is available, with all prespecified outcomes reported. | No unexpected missing data for the outcomes | No other significant biases |
| PERSEUS  2024 | The specific randomization methodology and tools utilized were not delineated. | The specific randomization methodology and tools utilized were not delineated. | Open-label | Open-label | The study protocol is available, with all prespecified outcomes reported. | No unexpected missing data for the outcomes | No other significant biases |

Supplementary Table 2. MRD assessment in the 5 RCTs included in the analysis of MRD negativity.

| Clinical trials | Time point | Sample source | Methods | Sensitivity |
| --- | --- | --- | --- | --- |
| CASSIOPEIA 2019 | Patients who achieved a VGPR or better at 6, 12, and 24 months of maintenance or observation. | Bone marrow aspirate | NGS and flow cytometry | 10^-5^ |
| CEPHEUS 2025 | At the time of suspected CR and at 12, 18, 24, 30 and 36 months after the first dose and annually thereafter in patients who achieved a confirmed CR. | Bone marrow aspirate | NGS | 10^-5^ |
| GMMG-HD7 2022 | Post-transplant | Bone marrow aspirate | Flow cytometry | 10^-5^ |
| GRIFFIN 2020 | At the end of the study. | Bone marrow aspirate | NGS | 10^-5^ |
| PERSEUS 2024 | At a CR or better at any time after the date of randomization during the study. | Bone marrow aspirate | NGS | 10^-5^ |

VGPR: very good partial response. NGS: next-generation sequencing. CR: complete response.

Supplementary Table 3. Sensitivity analyses for the rate of negative status for MRD by excluding each study.

| Excluded studies | P value | OR (95 %CI) | I^2^ (%) | Heterogeneity (P value) |
| --- | --- | --- | --- | --- |
| CASSIOPEIA 2019 | 0.002 | 1.86 (1.25, 2.78) | 21% | 0.28 |
| CEPHEUS 2025 | ＜0.0001 | 2.28 (1.56, 3.34) | 0% | 0.85 |
| GMMG-HD7 2022 | 0.0003 | 2.11 (1.41, 3.16) | 22% | 0.24 |
| GRIFFIN 2020 | 0.0008 | 1.94 (1.32, 2.85) | 21% | 0.24 |
| PERSEUS 2024 | 0.004 | 1.87 (1.23, 2.86) | 19% | 0.25 |

Supplementary Table 4. Sensitivity analyses for the rate of negative status for MRD by excluding specific studies.

| Excluded studies | P value | OR (95 %CI) | I^2^ (%) | Heterogeneity (P value) |
| --- | --- | --- | --- | --- |
| Studies not exclusively using NGS for MRD detection (CASSIOPEIA 2019 and GMMG-HD7 2022) | 0.007 | 1.93 (1.20, 3.09) | 46% | 0.16 |

NGS: next-generation sequencing. MRD: minimal residual disease.

Supplementary Table 5. Sensitivity analyses for PFS by excluding each study.

| Excluded studies | P value | HR (95 %CI) | I^2^ (%) | Heterogeneity (P value) |
| --- | --- | --- | --- | --- |
| ALCYONE 2018 | 0.02 | 0.74 (0.57, 0.95) | 0% | 0.48 |
| AMaRC 03-16 2024 | 0.02 | 0.74 (0.59, 0.95) | 0% | 0.48 |
| CASSIOPEIA 2019 | 0.03 | 0.75 (0.59, 0.97) | 0% | 0.49 |
| CEPHEUS 2025 | 0.01 | 0.73 (0.57, 0.93) | 0% | 0.50 |
| GMMG-HD7 2022 | 0.004 | 0.68 (0.53, 0.88) | 0% | 0.75 |
| GRIFFIN 2020 | 0.04 | 0.77 (0.61, 0.98) | 0% | 0.71 |
| IMROZ 2024 | 0.009 | 0.72 (0.56, 0.92) | 0% | 0.55 |
| OCTANS 2023 | 0.02 | 0.76 (0.60, 0.96) | 0% | 0.63 |
| PERSEUS 2024 | 0.07 | 0.79 (0.61, 1.02) | 0% | 0.58 |

Supplementary Table 6. Sensitivity analyses for PFS by excluding specific studies.

| Excluded studies | P value | HR (95 %CI) | I^2^ (%) | Heterogeneity (P value) |
| --- | --- | --- | --- | --- |
| Phase 2 studies (AMaRC 03-16 2024 and GRIFFIN 2020) | 0.04 | 0.78 (0.61, 0.99) | 0% | 0.60 |
| Non ImiDs backbones studies (ALCYONE 2018, AMaRC 03-16 2024, and OCTANS 2023) | 0.02 | 0.76 (0.58, 0.99) | 4% | 0.39 |

ImiDs: immunomodulatory drugs.
